# Perceptual-Motor Abilities and Reversal Frequency of Letters and Numbers in Children Diagnosed with Poor Reading Skills

**DOI:** 10.3390/bioengineering11121197

**Published:** 2024-11-27

**Authors:** Danjela Ibrahimi, Marcos Aviles, Juvenal Rodríguez-Reséndiz

**Affiliations:** 1 Facultad de Medicina, Universidad Autónoma de Querétaro, Santiago de Querétaro 76010, Mexico; danjela.ibrahimi@uaq.mx; 2 Brain Vision & Learning Center, Santiago de Querétaro, Misión de Capistrano 117, Juriquilla 76226, Mexico; 3 Facultad de Ingeniería, Universidad Autónoma de Querétaro, Santiago de Querétaro 76010, Mexico

**Keywords:** ocular diseases, development eye movement test, eye movement pattern, academic learning, perceptual–motor skills

## Abstract

Purpose: This paper investigated the visual–perceptual and visual–motor skills and the reversal frequency of letters and numbers that mirror one another in one hundred children aged 6–13 years diagnosed with poor reading skills. Methods: TVPS-4th, VMI-6th, and RFT were performed. Age and sex analysis was carried out. The impact of the eye movement patterns in the perceptual–motor skills and laterality–directionality concepts was also estimated to determine the relationship among tests to predict future results. Results: Most children scored between average and 3 stds below average on the motor VMI-6th test, while half of the participants scored between average and 2 stds below average on TVPS-4th. In the RFT, the majority scored between average and 1.5 stds below average. Participants scored higher on the spatial relationship subtest of the TVPS-4th and lower in the VMI-6th test (p<0.001). Statistically significant differences were found between the youngest and oldest participants on the TVPS-4th overall performance, as well as VD, FC and VFG skills (p<0.05). A strong relationship was found between the TVPS-4th and VMI-6th, (p<0.001). RFT results were different among all groups (p<0.05). The RFT was better related to the VMI-6th than TVPS-4th; however, it was statistically insignificant. The horizontal component of the DEM test was the best predictor for the TVPS-4th and ratio for the RFT, without attaining statistical significance. No sex differences were found. Conclusions: Results showed that children with poor reading skills exhibit perceptual–motor and reversal frequency difficulties, which are independent of the oculomotor performance. Considering that visual and motor processing are essential elements of the reading and writing process, their evaluation and treatment should be included as part of the multidisciplinary approach for children with poor reading skills. This would boost the general outcome and contribute to their academic achievement.

## 1. Introduction

In a previous article, the eye movement patterns of children diagnosed with poor reading skills were analyzed [1]. This research focuses on their visual–perceptual and visual–motor skills, as well as the reversal frequency of letters and numbers that are mirror images of one another, being part of the visual processing system. Our goal was to estimate their perceptual–motor abilities and laterality–directionality skills and determine the relationship among tests to predict future results, so children with poor reading skills can be referred for further evaluations and treatments to enhance their academic learning.

The cognitive ability model considers visual processing a domain-specific factor [2], which allows the use of simulated mental images to solve problems [3], and the learning skills pyramid places visual information processing lower in the hierarchy than reading and writing [4].

Therefore, children should be able to address visual–perceptual and motor skills correctly before the reading and writing process begins. It has been documented that developmental disorders, visual dysfunctions, learning difficulties, etc., place children at higher risk for problems with visual–perceptual and visual–motor skills [5,6,7,8], which correlate with their difficulties on academic tasks and activity of daily living [9,10]. The contribution of visual–perceptual skills to the reading process is not fully understood; however, it has been hypothesized that visual–spatial mechanisms support the growth of visual coding of print (orthography) [11], and the development of phonological awareness is partially dependent upon the normal input from the visual system [12]. The literature shows that several people with dyslexia have a visual processing deficit independent of phonological skills [13] as well as a visual–attentional deficit [14]. On the other hand, visual–motor skills play an essential role in activities such as reaching, grasping, handwriting, reading, etc. [15]. It is estimated that up to 15% of school-age children experience problems with visual–motor skills [16], impacting their performance on reading decoding tasks, as gross motor skills are reported to be implicated in reading acquisition [17] and children’s academic abilities in language, and mathematics [18]. In addition, the ability to copy letters accurately has been associated with the performance of visual–motor skills. Therefore, letter reversals and subsequent reading disorders are at least partially related to a disordered visual processing system [19].

Research has shown that children with neurologically based reading disabilities exhibit greater reversal frequency [20] than typically developed children, which persist with age [21]; therefore, there is a need for its evaluation in school-age children with poor reading skills. To the best of our knowledge, it is the first time that a complete battery of neuro-optometric tests, which assesses visual–perceptual abilities, visual–motor skills, and laterality–directionality aspects, has been used to determine the performance of school-age children diagnosed as “poor readers”.

In children, poor reading ability is defined by neuropsychologists as a score of below one standard deviation in two or more of the evaluated skills (precision, fluency, and reading comprehension), while in dyslexia or learning disabilities, a score of below two standard deviations [22]. However, there is important research focused on children with dyslexia [23,24,25], ADHD [25], learning disabilities [26], developmental coordination disorders [17], sensory integration issues [27] etc., specific research on poor readers is lack. For the assessment, the Test of Visual Perceptual Skills 4th ed. (TVPS-4th) [28], Visual–Motor Integration Test 6th ed. (VMI-6th) [29], and Reversal Frequency Test of Gardner (RFT) [23] were used as they are well-standardized and globally accepted by the scientific community in the field of neuro-optometry.

An accurate evaluation and diagnosis are considered the first step toward a successful treatment in clinical practice.

There currently needs to be more research on the visual processing of children and whether or not they face reading challenges. Although neuropsychological assessments are common, the evaluation of vision as a neurological function is typically overlooked. Consequently, most children are only referred for neuro-optometric evaluation after being treated as patients with learning difficulties, often without achieving the expected results. Integrating the assessment of the visual system and its components into the multidisciplinary approach for children with poor reading skills could significantly reduce time, effort, and costs while also preventing unnecessary frustration.

This research explored patterns and relationships among tests to predict future results on the visual processing of children with poor reading skills and help them through this process, considered an essential milestone for academic learning [30]. Likewise, being neuro—an optometrist is the first filter for patients with visual dysfunctions and visual processing information deficiencies. Hence, visual health professionals’ assessment of children struggling with reading becomes relevant. Therefore, excluding the implication of any visual components related to their academic achievement before being treated as patients with learning difficulties becomes a priority.

This paper is structured as follows: In Section 2, the methodologies of the different analyses performed are described. In Section 3, the results are presented. In Section 4, the results are discussed. Finally, in Section 5, the conclusions are given.

## 2. Materials and Methods

This descriptive, transversal, and prospective study included 100 children diagnosed as poor readers by their neuropsychological department. Data on medical history and clinical examinations were collected at the Brain Vision and Learning Center, a research center, in collaboration with the Autonomous University of Querétaro, Mexico, from June to December 2023. The study conformed to the principles of the Declaration of Helsinki. Consent from the participants and parents was obtained before performing any procedure.

The clinical examination included the following aspects of the visual processing system: the visual efficacy exam (included an assessment of ocular health to exclude pathologies or structural damages), oculomotor performance (saccades and pursuits), visual–perceptual and visual–motor skills, and reversals frequency of letters and numbers. In this research, the last three areas of the visual assessment were statistically analyzed, as results obtained from the analysis of the first two areas have already been published in a previous paper [1].

The number of patients who came to the center and met the inclusion criteria determined the size of the sample. Eligibility was established based on the following inclusion/exclusion criteria:

- Inclusion criteria: visually typical children (VA≥0.1LogMar); aged 6 to 13 years; without ocular pathologies or previous visual treatments (except the use of their prescription); similar economic status; average IQ score for their chronological age. The age limit for participants was 13 years, as the target population of the DEM test used in the previous paper was children aged between 6 and 13.11 years.
- Exclusion criteria: diagnosis of strabismus/amblyopia; the presence of conditions such as attention-deficit/hyperactivity disorder, epilepsy, dyslexia, and depression; use of medications that affect the central nervous system; and premature birth. Poor reading ability is defined by neuropsychological assessments “as a score of below one standard deviation in two or more of the evaluated skills (precision, fluency, and reading comprehension), while dyslexia or learning disabilities, a score of below two standard deviations” [22].

In the field of neuro-optometry, the assessment of visual performance is divided into three important blocks: the sensorimotor balance of the visual system, which determines the binocular state of a child; oculomotricity to assess spatial and temporal localization; and the analysis of visual–perceptual, visual–motor and laterality–directionality skills to determine processing and motor pattern conversion abilities (being the third block, the scope of the research). The revised versions of the TVPS-4th, VMI-6th and Reversal Frequency Test of Gardner were used for this study [31,32].

The evaluation took place over three consecutive days (45 min sessions). Dr. Danjela Ibrahimi, Ph.D. in Optometry and highly qualified in this area, conducted the assessments. Evaluations were performed in the morning to ensure reliable data.

- First day: Sensorimotor balance of the visual system and oculomotricity.
- Second day: Analysis of the visual–perceptual skills.
- Third day: Evaluation of the visual–motor skills and laterality–directionality concepts.

Results of the sensorimotor balance and oculomotricity were published in our previous paper. This paper focused on visual–perceptual and motor skills and laterality–directionality concepts. The test used to evaluate the skills mentioned above.

### 2.1. Evaluation of Visual–Perceptual Skills (VPSs) and Visual–Motor Skills (VMSs) Using the TVPS-4th and VMI-6th

TVPS-4th [33] and VMI-6th [34] were administered, and raw scores were used to ultimately determine scaled scores, standard scores, percentiles, and perceptual ages. TVPS-4th evaluates the following seven areas: visual discrimination, visual memory, spatial relationships, form constancy, sequential memory, figure-ground, and visual closure. Scaled scores were used to analyze each subtest (with a mean value of 10 and std of 3), and standard scores were used to analyze the overall performance as determined in the literature (mean value of 100 and std of 15). Motor VMI-6th was chosen for analysis as it is directly related to fine-hand motor coordination. For statistical analysis and the purpose of this study, raw scores were then converted into scaled and standard scores. Scores of one standard deviation below the mean and above are considered the norm (the patient should obtain at least a standard score of 85 or a percentile rank of 20%).

The scoring procedure for the TVPS-4th and VMI-6th is the same. No distinction is made between male and female participants, and all steps are protocolized. The process is divided into four basic steps as summarized below.

- The first step is to calculate the perceptual age, which derives from the date of the test and birth date.
  − Date of test: 13 November 2024;
  − Birth date: 10 October 2024;
  − Difference among them: 6-01-03 (6 years, 1 month and 3 days = 6 years, 1 month).
- The second step is to convert raw scores to scaled scores, using the provided tables.
- The third step is to convert scaled scores to percentile ranks.
- For the overall performance, scaled scores of the seven subtests are summed up and converted to standard scores. After that, standard scores are converted to percentile ranks.

### 2.2. The Reversal Frequency Test of Gardner (RFT)

This test was used to measure the reversal frequency of letters and numbers that are mirror images of each other. The recognition subtest enables the child to choose between the correctly oriented letter or number and those presented as mirror images. Raw scores (number of errors) were then converted to percentiles as recommended by the literature [35]. A percentile rank from one standard deviation and above is considered the norm (from 20% and above).

The RFT is similar to the other two tests, as it contains percentile ranks which are useful to be understood by parents and teachers. However, here, gender differences are made. The test is composed of three subparts: the first is the execution of letters and numbers; the second, which was used in this paper, is about the reversal frequency of letters and numbers; and the third is the matching part. The RFT is a well-established, standardized and frequently used test by optometrists all over the world. The test has been used in the field of research by Dr. Gardner and proved itself a useful tool to analyze children with and without learning difficulties [36].

Considering that this group of poor readers was previously found to exhibit oculomotor difficulties, the impact of the eye movement patterns on their perceptual–motor skills and laterality–directionality concepts was also estimated. Exploring their relationship helps prevent and boost treatments in children with poor reading skills. Oculomotricity was evaluated using The Development Eye Movement test.

### 2.3. The Development Eye Movement Test (DEM)

The oculomotor assessment, which explores the saccadic eye movement ability of a child, is carried out using the psychometric DEM test [37]. Vertical and horizontal eye movements are analyzed. The time required to complete each subtest and the number of errors (omission, addition, substitution, and transposition) are recorded. The vertical subtest is mainly related to the child’s automaticity skills, while the horizontal part is related to oculomotor abilities. The ratio is obtained by dividing the adjusted horizontal time by the vertical time. The ratio directly compares the vertical (automaticity) and horizontal (oculomotor control + automaticity) test performance results. Standard scores and percentile ranks are used to characterize the eye movement patterns of patients.

More detailed information on the DEM test, sample and methods used in this research is found in a previous article, as this paper continues the other [1].

### 2.4. Statistical Analysis

The One-Way ANOVA was chosen for statistical analysis as it is used when the means of three or more groups are compared. This test tells whether the sample provides sufficient evidence to conclude that the groups’ population means are different.

Simple linear regression was used to estimate the relationship between two quantitative variables (how strong this relationship is, as well as the value of the dependent variable at a certain value of the independent variable), while in the multiple regression analysis, a criterion variable is predicted by two or more predictor variables.

## 3. Results

In this section, the results obtained are presented and examined, highlighting the key findings and any relevant patterns observed.

### 3.1. Descriptive Statistics

This work is a descriptive, transversal and prospective study. One hundred children, diagnosed as “poor readers” with a mean age of 8.0±1.8 years, participated in this study, of whom 64 were male (64%) and 36 were female (36%). From the total, 86 (86%) were exophoric (mean value 8.2±4.2), 14 (14%) were esophoric (mean value 3.6±2.4), and the mean degree of stereopsis was 27.5±10.2. Visual acuity was ≥0.1logMar. The TVPS-4th, VMI-6th, and RFT were submitted to all participants, and the results were compared between them and with the DEM test.

### 3.2. Characterizes Patients Behavior Patterns in the TVPS-4th, VMI-6th and RFT

**Figure 1:**
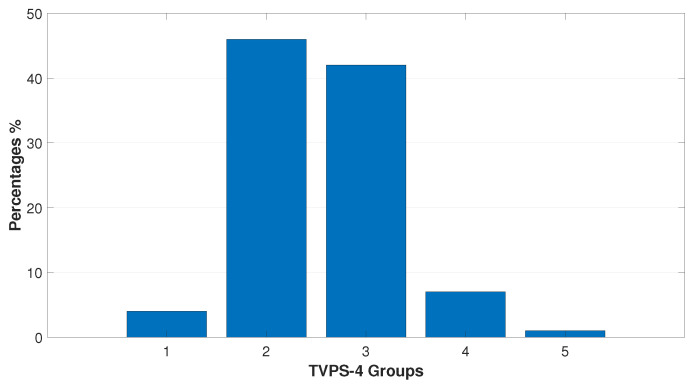
Standard scores (SSs) of the overall performance on the TVPS-4th and the percentages of its distribution into five percentile ranks as follows: group 1 (4%), 70–84 ss; group 2 (46%), 85–100 ss; group 3 (42%), 101–115 ss; group 4 (7%), 116–130 ss; and group 5 (1%), 131–145 ss. From the chart, it is seen that 88% of the participants lie between group 2 (from 1 std below to average performance) and group 3 (from average performance to 1 std above) being these results are considered within the norm for their age.

**Figure 2:**
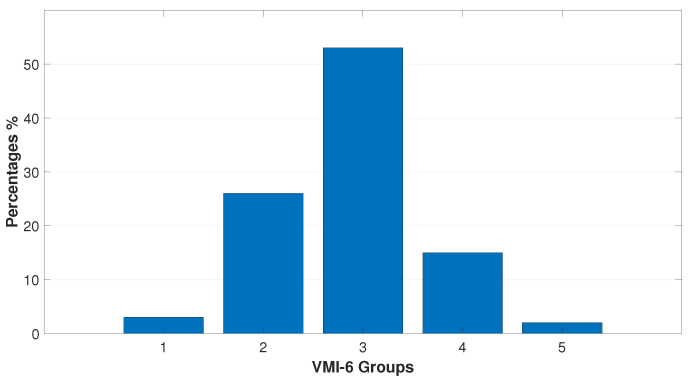
Illustrates the standard scores (SSs) obtained on the motor VMI-6th test and the percentages of its distribution into five percentile ranks as follows: group 1 (3%), 55–70 ss; group 2 (26%), 71–84 ss; group 3 (53%), 85–100 ss; group 4 (15%), 101–115 ss; and group 5 (2%), 116–130 ss. One datum was excluded as it fell 3 stds below the mean. From the chart, it is seen that 78% of the participants lie between groups 2 and 3 (between average and 2 stds below average), and only 17% of them are considered above average.

**Figure 3:**
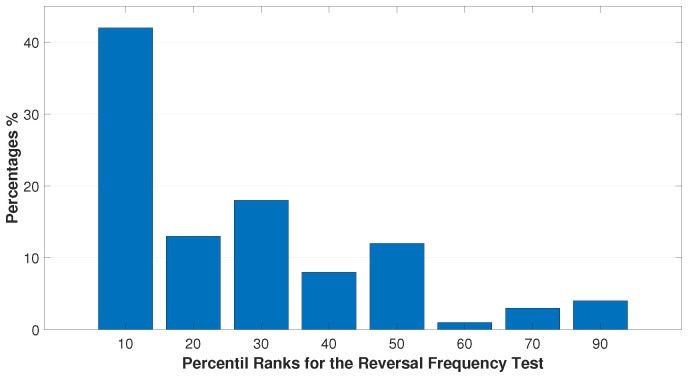
Distribution (%) sorted into different percentile ranks of the RFT. From the chart, it is seen that 42% of poor readers were classified as 1.5 stds below the mean, whereas 38% lay between average and 1 std below average. Additionally, 12% of them showed an average performance, and only 8% were considered above average.

Figure 1, Figure 2 and Figure 3 represent the data distribution (%) of the submitted tests into their respective percentile ranks.

Lower values and more heterogeneous data were obtained on the Visual–Motor Integration Test (VMI-6th), with mean value (90.14±13.18) vs. (101.23±9.83) on the Visual–Perceptual Test (TVPS-4th). Additionally, 82% of children scored between average and 3 stds below average on the motor VMI-6th test, whereas 50% of participants scored between average and 2 stds below average on the TVPS-4th. No child scored 3 stds below average on the TVPS-4th.

Figure 3 shows that 80% of children with poor reading skills scored between average and 1.5 stds below average, a significant result when the general frame of a child’s academic performance is considered. A failure follows their reading difficulties in their reversal recognition skills. As a result, they miss identifying the correct directionality of letters and numbers during reading or writing, which is a characteristic of children with dyslexia.

### 3.3. Section II

This section describes the results obtained on each test and the deviations from the mean values.

Table 1 shows that the highest scores were obtained in the spatial relationship subpart of the TVPS-4th, and the lowest one, in sequential memory. Likewise, participants scored better in the TVPS-4th than the VMI-6th.

The One-Sample T-test was used to see the differences between the sample and the established values. For the TVPS-4th and VMI-6th, the mean value is set to 100, while for each subtest of the TVPS-4th, the mean value is 10. Statistically significant differences from the mean value were found for the spatial relationships and VMI-6th. Results are presented in Table 2.

As shown in Table 2, the spatial relationship subtest of the TVPS-4th and the performance on the VMI-6th test were the only two variables of interest. Participants obtained higher values on the first one and lower values on the second one when compared to the normative data.

Considering these data, age and sex analyses were performed to find any differences or similarities in the scores obtained through these assessments.

### 3.4. Age Group Analysis

Patients were divided into three groups based on their reading, writing, and comprehension levels, as well as the time spent in front of digital screens and performing near-distance tasks. This study builds upon our recently published article on the oculomotor patterns of children diagnosed with poor reading skills. Moreover, previous studies have demonstrated that these cohorts are useful for analyzing visual–cognitive abilities in children, as they exhibit similar performance patterns [38,39,40,41,42].

- Group 1 (47 children): from 6.0 to 7.11 years;
- Group 2 (29 children): from 8.0 to 9.11 years;
- Group 3 (24 children): form 10.0–13.11 years.

Visual–perceptual skills, visual–motor integration abilities, and reversal recognition errors were compared among groups. The One-Way ANOVA with Tukey HSD for multiple comparisons was performed. The statistical analysis results are presented in Table 3 and Table 4. The homogeneity of variances showed unequal results only for sequential memory.

As depicted by Table 3, statistically significant differences were obtained for the visual discrimination subtest, form constancy, visual figure-ground, the overall performance on the TVPS-4th, as well as the RFT. More specifically, Table 4 represents the mean values, stds and *p*-values of the variables of interest.

Table 4 shows that differences among groups are observed when the youngest is compared to the oldest (1 vs. 3), and only for the following perceptual abilities: visual discrimination, form constancy, and visual figure-ground. The overall performance on the TVPS-4th was age-dependent, whereas all groups obtained similar scores on the VMI 6th test. The RFT showed statistically significant differences among groups, with the number of committed errors being age-dependent.

Table 5 presents the statistical analysis and relationship among the TVPS-4th, VMI-6th, and RFT, as well as the implication of the DEM test on these results.

The statistical analysis did not reveal sex differences. Male and female participants obtained similar results in all tests.

In phase two, the effect of the oculomotor performance and its components on the visual–perceptual, visual–motor, and reversal recognition abilities was analyzed. Oculomotor performance was considered the independent variable based on the hierarchy of the visual system; any further analysis of the visual information requires good eye movements. The visual–perceptual overall performance, visual–motor integration skills, and reversal recognition abilities were compared to the vertical, horizontal, and ratio results. Multiple linear regression analysis was performed to identify the impact of the DEM results on perceptual–motor skills and to describe the relationship among variables to predict future results. Standard scores were used to analyze the relationship among the D.E.M, TVPS-4th and VMI-6th test, whereas percentile ranks for the RFT.

The multiple regression analysis showed that the components of oculomotor performance do not predict the result on the TVPS-4th, VMI-6th, or RFT. However, a better predictability was found for the data obtained on the TVPS-4th compared to the other two tests. These results are seen in Table 5.

To see which component of the DEM test can better predict the results on the TVPS-4th, the standardized coefficient beta, *p*-value, and partial correlations were considered.

Data showed that the horizontal performance was the only independent variable that could better predict the results on the TVPS-4th, where p=0.197, beta=0.356, and partial correlations (0.127). Despite this weak correlation, these results provide relevant information if the child in the school’s environment context is considered. The horizontal eye movements represent how we read; therefore, a probable relationship between how they read and the process emerges to help teachers derive these children for further evaluation and treatment to improve academic achievements. For the RFT, the strongest predictor was the DEM ratio (differentiates the effect of automaticity on the oculomotor performance), where p=0.053, *beta* = 0.380, and partial correlation =0.196. The standardized coefficient beta, *p*-value, and partial correlations were even more insignificant for the VMI-6th.

Finally, the simple linear regression analysis was performed to identify any predictors among the TVPS-4th, VMI-6th, and RFT (percentile ranks obtained on the three tests were used for the purpose). Respecting the hierarchy of the visual system, the visual–perceptual analysis comes before the ability to draw and recognize the directionality of the written letter. Therefore, the TVPS-4th overall performance was the independent variable.

**Figure 4:**
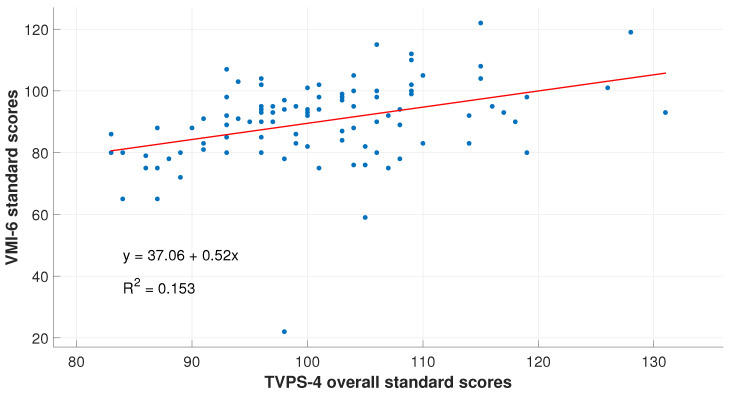
Relationship between the data obtained on the TVPS-4th and VMI-6th. As seen, results obtained by poor readers on the visual–perceptual and visual–motor skills are co-dependent and predict each other, with an R2 linear value of 0.153. From the graph, we predict that a lower/higher performance on the perceptual analysis would directly impact its motor expression.

For the RFT as a dependent variable of the TVPS-4th, the model summary showed an Adjusted R Square =0.018, *F*-value =2.76 and p=0.099. A stronger relationship was found between the RFT and VMI-6th, where Adjusted R Square =0.025, *F*-value =3.551, and p=0.062. However, statistically significant results were obtained only between the TVPS-4th and VMI-6th, with Adjusted R Square =0.144, *F*-value =17.702 and p<0.001. This relationship is illustrated in Figure 4.

## 4. Discussion

This study investigates the visual–perceptual, visual–motor, and reversal frequency skills of children diagnosed as poor readers and the interaction among tests to predict future results so children with poor reading skills can be referred for further evaluations and treatments to enhance their academic achievements. Considering that this group of poor readers was previously found to exhibit oculomotor difficulties, the impact of the eye movement patterns on their perceptual–motor skills and laterality–directionality concepts was also estimated.

To the best of our knowledge, it is the first time that the interaction among these essential elements of visual processing is explored, and the literature shows no studies assessing the perceptual–motor abilities and directionality concepts of children with poor reading skills. It is important to distinguish the differences among poor readers (PDs), children with developmental dyslexia (DD), and learning disabilities (LDs) to understand the differences and/or similarities between this study and what is found in the literature. In children, poor reading ability is defined by neuropsychologists as “a score of below one standard deviation in two or more of the evaluated skills (precision, fluency, and reading comprehension), while in dyslexia or learning disabilities, a score of below two standard deviations” [22]. Knowledge of reading difficulties comes from studies in patients with development dyslexia. Therefore, it is only natural to understand the study’s results based on the existing literature. In past decades, researchers have supported the hypothesis of M pathway (transient system) deficit in dyslexia [43], related to the concept of “where” things are located in space. Nevertheless, during this last decade, different studies have hypothesized a combined impairment of both M- and P pathway (sustained system, associated with the concept of “what” are the things I am looking at) in dyslexia, which may better explain the visual deficits found in these patients [44,45]. Therefore, a more in-depth analysis of children with reading challenges should be implemented to assess visual and motor processing areas based on the knowledge of the P and M pathway.

Firstly, the statistical analysis suggested that eye movement patterns did not impact the results obtained on the rest of the neuro-optometric assessment (TVPS-4th, VMI-6th and RFT). Nonetheless, the horizontal subpart of the DEM test and ratio were the two components that best related to the TVPS-4th and RFT without reaching statistical significance. These data suggest that neurological pathways for oculomotor and perceptual abilities activate different brain areas, as seen by fMRI or EEG studies [46,47]. Perceptual learning is mostly related to higher cortical areas other than just V1 [48,49], which makes us hypothesize that performance in visual–perceptual areas may not be reflected by the oculomotor behavior of poor readers, as supported by a recent study in dyslexic children [23]. Nevertheless, a link between visual–attentional processes and oculomotor performance in children with dyslexia has been found, which impacts the reading outcome [14]. Likewise, the horizontal and vertical subpart of the DEM test has been shown to predict reading outcomes in healthy kindergarten [30]. However, we do not completely understand their interaction with perceptual–motor skills and visual–spatial abilities (laterality, directionality, and relative position in space) in children presenting reading difficulties (related to a developmental condition or not). When considering that 84% of poor readers exhibit oculomotor difficulties [1], it becomes crucial to raise awareness among professionals regarding the role of eye movements and perceptual–motor skills in school-age children. Children with reading difficulties should be referred for more in-depth visual assessment to exclude its implication in their academic struggle.

Secondly, poor readers scored lower on the VMI-6th test (most of the participants scored between average and 3 stds below average), which means that not only phonological aspect but also a good development of visual–motor integration skills is necessary to decode reading successfully. This relationship is explained when analyzing the effect of learning letters by handwriting in preschool children and their reading skills [50]. Furthermore, fine motor skills like the one needed during the VMI assessment can predict achievement in reading at the end of first grade [18]. From these studies, it has been hypothesized that the process of learning letters is based on a multimodal representation of sensory and motor skills, which has been corroborated in more recent studies conducted in children with DD [24] and LD [26].

If we go back to the learning pyramid, writing comes after perception, and additional cortical areas, which are not involved during the perceptual analysis, are needed. Brain regions for visualization, motor planning, and fine motor skills graphically represent any information provided through the visual system. Writing is a more complex neurological process than perception; thus, struggling could be more frequent. Nevertheless, perceptual learning has been used to enhance motor skills. Therefore, possessing good visual–perceptual skills could be essential to visual–motor integration abilities [51].

Likewise, even though it did not reach statistical significance, a stronger relationship was found between the RFT and VMI-6th than TVPS-4th performance, suggesting that the motor component is of greater significance for the process of directionality than the perceptual analysis. From a clinical perspective, the data have important implications when the position of a child with poor reading skills in an academic environment is considered, with change as the only constant, and they do not seem to keep up the pace. It becomes clear that assessing visual–motor integration skills becomes essential to avoid later frustration, as reading interacts actively with fine motor skills.

When exploring their visual–perceptual skills, almost half of the sample scored between average and 2 stds below average. This information points out how children with poor reading skills may be at risk for visual–perceptual deficits. As specialists caring for children struggling academically, we would like them to be above average so they compensate for the increasing academic load as they grow. Research on children with DD [24] or LD [26] has shown that part of their difficulties is related to their sensory deficits (visual, motor, or auditory). This group of patients presents reduced visual–perceptual and motor abilities as part of abnormalities in the magno and/or parvocellular pathway [44,45].

The “M pathway” conveys information related to the perception of movement, depth, and large-scale objects, providing a rapid and transient response. In contrast, the “P pathway” transmits information about the perception of color, form, and fine details, offering a slower but sustained response.

In simpler terms, the “M pathway” answers the question “Where is the object of interest located in space?” Meanwhile, the “P pathway” addresses “What are the details of this object?” [52].

This model should apply to any neurodevelopmental disorder followed by sensorimotor deficit, such as reduced fine motor skills or perceptual deficiencies. Based on the level and specificity of the disorder, heterogeneous perceptual and motor manifestations are explained [53].

Interestingly, participants scored higher in spatial relationships than the other evaluated skills on the TVPS-4th. Considering that recognition of shapes found in nature (such as circles, squares, triangles, etc) and their specific place to be integrated into the whole picture are considered the basis of the learning process, we believe these results are the reason. Likewise, even in dyslexic children, spatial relationships dominate the rest of perceptual skills [23]. As neuroscience explains, abilities integrated from an early age are easier to persist over time.

Visual–perceptual and motor skills were found to be strongly correlated, suggesting they can predict each other’s results. If we take into consideration that graphomotor skills depend on the processing and analysis of visual information, results of the TVPS-4th (analysis of the visual information) and VMI-6th (graphomotor reproduction of this information) were expected to be related.

Additionally, the RFT showed that 80% of children with poor reading skills scored between average and 1.5 stds below average, which is significant when the general frame of a child’s academic performance is considered. A failure follows their reading difficulties in their reversal recognition skills. As a result, they miss identifying the correct directionality of letters and numbers during reading or writing, which is a characteristic of children with dyslexia [23].

It is well known that the consolidation process of the laterality–directionality concepts needed to understand reversal letters such as b-d or p-q takes place during the first academic years. Nevertheless, the literature suggests that directionality may take longer to be completely dominated; thus, younger children are prone to commit more errors than older ones during the reading and writing process [54]. Failures in these aspects of learning are very common among children with reading difficulties [55] and dyslexia [23].

Ultimately, age and sex analyses were performed, proving age-dependent. More specifically, the visual–perceptual analysis and reversal frequency recognition abilities were more strongly related to age than visual–motor skills. These support the theory of autonomous systems of motor-reduced visual perception and visual–motor integration skills [56].

What seemed interesting, though, was that the youngest group scored higher than the oldest group. These results are more related to the number of errors permitted during the first years of the learning process, as it is still being consolidated. Nevertheless, the only study to use the TVPS-R (a much older version of the test than the one used in this paper) to evaluate the visual–perceptual skills of dyslexic children showed that after a perceptual training of ten weeks, visual discrimination, form constancy, figure-ground, and TVPS overall scores improved after the training process. Interestingly, in this paper, these were the only areas of the TVPS-4th to be age-dependent [23].

These results do not come as a surprise as research has shown that different components of the visual processing system such as oculomotricity [57,58], spatial attention [59], perceptual processing of visual objects [60], acquisition of fine motor skills [61], etc., are age-related. The sample observed differences among groups when the youngest group was compared to the oldest group. It is well known that cognitive development is associated with age-related enhancements across several perceptual–motor domains, as younger children perform worse than older ones in most aspects [62]. Nonetheless, as children grow older, giving them the right tools at an early stage can compensate for their difficulties, as cortical plasticity is age-related, and critical periods have the most significant impacts on the neuronal brain network [63].

Considering that a significant portion of information, especially among school-age children, is acquired through the visual system, its analysis becomes essential, being regarded as the first step toward further cortical processing.

Nevertheless, when children face academic challenges, the visual system is often the last aspect to be evaluated. Even when it is assessed, patients are typically referred to an ophthalmologist for standard visual examinations focusing on visual acuity, refraction, and general ocular health. However, critical areas related to the broader analysis of vision such as perceptual–motor abilities, sensorimotor balance, laterality directionality concepts, and oculomotor skills are frequently overlooked. Consequently, reading dysfunctions stemming from poor visual performance may go unnoticed. A thorough analysis of vision, beyond mere visual acuity, is therefore crucial.

Without a comprehensive evaluation of vision as a complex neurological process, poor reading abilities will continue to be misclassified as general learning problems, even in the absence of a specific neuropsychological diagnosis. This misclassification will persist unless the root causes of these disorders are properly identified and addressed. To enhance overall outcomes and support academic achievement, the assessment and treatment of visual information processing should be integrated into the multidisciplinary approach for children with poor reading skills.

This study identified the strongest and weakest aspects of visual–perceptual and visual–motor abilities and reversal frequency recognition skills in poor readers. Even though these children are classified as only 1 std below the average in their reading abilities (unlike children with dyslexia or learning disabilities who score below 2 stds), the statistical analysis showed significant deficiencies in their perceptual–motor abilities and directionality concepts.

Similarities among our sample, composed of children with poor reading skills and children with dyslexia and learning difficulties, make us hypothesize that the same cortical circuits could be affected at a different level. Nevertheless, the principal skills of the reading process are compromised and should be addressed in the same way to avoid academic struggle and future frustration. Poor readers should be considered, and exhaustive assessments, including the visual processing information, must be added to their multidisciplinary approach to enhance their academic life.

The data suggest that it is strongly recommended that school-age children who struggle with any aspect of reading should be referred for a more in-depth neuro-optometric assessment. This would help to identify the actual cause of their difficulties and exclude the implication of any visual component that impairs their academic achievement.

## 5. Conclusions

This paper explores the role of visual–perceptual and visual–motor skills, along with laterality–directionality concepts, in 100 children with poor reading skills. Our findings indicate that poor readers face challenges with perceptual–motor skills and reversal frequency, which appear to be independent of their oculomotor performance. Given that visual and motor processing are crucial components of reading and writing, their evaluation and intervention should be integral to the multidisciplinary approach for children with poor reading skills. Moreover, since age emerged as a key variable influencing the results, early intervention could significantly benefit these patients. Professionals should prioritize enhancing motor integration abilities and addressing reversal difficulties, as these are the most impacted areas. Focusing on these aspects of visual analysis could improve overall outcomes and contribute to the academic success of children struggling with reading.

## Figures and Tables

**Table 1:** Mean values and standard deviations for each subtest of the TVPS-4th, its overall performance, and the results obtained on the motor VMI-6th and RFT.

Analyzed Variable	Mean	Std.
N = 100		
Visual discrimination	9.9	2.9
Visual memory	10.1	3.0
Spatial relationships	11.8	3.3
Form constancy	10.1	3.4
Sequential memory	9.7	2.6
Visual figure-ground	10.4	3.7
Visual closure	9.9	2.9
TVPS-4 overall performance	101.2	9.8
VMI 6th	90.1	13.2
Reversals recognition errors	13.9	9.8

OSS: overall standard scores; SS: standard scores; Std.: standard deviations.

**Table 2:** Data obtained by the One-Sample T-Test analysis. It is seen that participants scored higher than the norm on the spatial relationship subtest and lower on the VMI-6th test. No other significant differences were obtained from the statistical analysis.

	*p*-Value	95% Confidence Interval of the Difference	
		Lower	Upper
Visual discrimination	0.641	−0.73	0.45
Visual memory	0.691	−0.48	0.72
Spatial relationships	<0.001	1.17	2.47
Form constancy	0.768	−0.57	0.77
Sequential memory	0.294	−0.78	0.24
Visual figure-ground	0.254	−0.31	1.15
Visual closure	0.893	−0.63	0.55
TVPS-4th OSS	0.214	−0.72	3.18
VMI-6th SS	<0.001	−12.48	−7.24

**Table 3:** The F and *p*-value of the One-Way ANOVA analysis for the visual–perceptual, visual–motor skills, and reversal recognition errors are presented. Statistically significant differences among groups were found for the visual discrimination, form constancy, visual figure-ground, overall performance on the TVPS-4th, and reversal recognition errors.

Analyzed Variable	One-Way ANOVA for Age Group Comparison	
	*F*-Value	*p*-Value
Visual discrimination	3.39	0.038
Visual memory	1.93	0.15
Spatial relationships	2.00	0.14
Form constancy	3.22	0.044
Sequential memory	1.28	0.282
Visual figure-ground	3.08	0.05
Visual closure	2.73	0.07
TVPS-4th OSS	5.32	0.006
Motor VMI SS	2.95	0.057
Reversal recognition errors	23.25	<0.001

**Table 4:** Shows the mean values and standard deviations of the variables of interest, as well as the *p*-value of the comparison among groups. Statistically significant differences were found between the youngest and oldest participants. RFT results were different among all groups.

Variables of Interest	Mean	Std. Deviation	*p*-Value
Visual discrimination			
1	10.53	3.44	
2	9.79	2.47	
3	8.63	2.24	0.029
Form constancy			
1	10.85	3.43	
2	10.00	2.79	
3	8.75	3.63	0.034
Visual figure-ground			
1	11.32	3.66	
2	9.97	3.25	
3	9.21	3.82	0.050
TVPS-4 OSS			
1	103.64	10.25	
2	101.69	7.95	
3	95.96	9.38	0.005
Reversals recognition errors			
1	19.30	8.61	(1 vs. 2) 0.001
2	11.83	8.51	(1 vs. 3) <0.001
3	5.79	6.49	(2 vs. 3) 0.023

OSS: overall standard scores. The mean difference is significant at the 0.05 level. Turkey HSD for multiple comparison based on the equality of variances.

**Table 5:** Results of the multiple regression analysis for the TVPS-4th, VMI-6th and RFT as dependent variables of the three components of the DEM test (horizontal, vertical and ratio SS). Data illustrate stronger predictability for the TVPS-4th.

Model	R Square	Adjusted R Square	F Change	Sig. F Change
TVPS-4th	0.077	0.048	2.676	0.051
VMI-6th	0.006	−0.025	0.184	0.907
RFT	0.040	0.010	1.318	0.273

## Data Availability

Database information is available upon request from the authors. The data are not publicly available due to privacy and ethical restrictions.

## Abbreviations

TVSP-4th	Test of Visual–Perceptual Skills, fourth edition
VMI-6th	Visual–Motor Integration Skills, sixth edition
RFT	Reversal Frequency Test of Gardner
DEM	Development Eye Movement Test
VD	Visual Discrimination Skills
FC	Form Constancy Skills
VFG	Visual Figure-Ground Skills
OSS	Overall Standard Scores
SS	Standard Scores
Std	Standard Deviation
